# The Marine-Derived Natural Product Epiloliolide Isolated from *Sargassum horneri* Regulates NLRP3 via PKA/CREB, Promoting Proliferation and Anti-Inflammatory Effects of Human Periodontal Ligament Cells

**DOI:** 10.3390/md19070388

**Published:** 2021-07-09

**Authors:** Eun-Nam Kim, Woguti Yvonne Nabende, Hyeyoon Jeong, Dongyup Hahn, Gil-Saeng Jeong

**Affiliations:** 1 College of Pharmacy, Keimyung University, 1095 Dalgubeol-daero, Daegu 42601, Korea; enkimpharm@gmail.com; 2 School of Food Science and Biotechnology, College of Agriculture and Life Sciences, Kyungpook National University, Daegu 41566, Korea; yvonwoguti@gmail.com (W.Y.N.); ddi02084@naver.com (H.J.); 3 Department of Integrative Biology, Kyungpook National University, Daegu 41566, Korea

**Keywords:** *Sargassum horneri*, periodontitis, protein kinase A (PKA), cAMP-responsive element-binding protein (CREB), epiloliolide

## Abstract

Currently, periodontitis treatment relies on surgical operations, anti-inflammatory agents, or antibiotics. However, these treatments cause pain and side effects, resulting in a poor prognosis. Therefore, in this study, we evaluated the impact of the compound epiloliolide isolated from *Sargassum horneri* on the recovery of inflammatory inhibitors and loss of periodontal ligaments, which are essential treatment strategies for periodontitis. Here, human periodontal ligament cells stimulated with PG-LPS were treated with the compound epiloliolide, isolated from *S. horneri.* In the results of this study, epiloliolide proved the anti-inflammatory effect, cell proliferation capacity, and differentiation potential of periodontal ligament cells into osteoblasts, through the regulation of the PKA/CREB signaling pathway. Epiloliolide effectively increased the proliferation and migration of human periodontal ligament cells without cytotoxicity and suppressed the protein expression of proinflammatory mediators and cytokines, such as iNOS, COX-2, TNF-α, IL-6, and IL-1β, by downregulating NLRP3 activated by PG-LPS. Epiloliolide also upregulated the phosphorylation of PKA/CREB proteins, which play an important role in cell growth and proliferation. It was confirmed that the anti-inflammatory effect in PG-LPS-stimulated large cells was due to the regulation of PKA/CREB signaling. We suggest that epiloliolide could serve as a potential novel therapeutic agent for periodontitis by inhibiting inflammation and restoring the loss of periodontal tissue.

## 1. Introduction

Chronic periodontitis causes tooth loss due to the loss of attachment of periodontal ligament tissue due to an inflammatory reaction, therefore restoration and reconstruction of the periodontal ligament through inhibition of periodontal inflammation are important in the treatment of periodontitis [1,2]. Lipopolysaccharide (LPS), a prototype class of PAMP3, is a component of the outer cell membrane of Gram-negative bacteria, and *Porphyromonas gingivalis* lipopolysaccharide (PG-LPS), which is capable of destroying the periodontal ligament during cell damage caused by periodontitis, contributing to the onset of periodontitis [3]. Human periodontal ligament (HPDL) cells play a role in connecting the roots of teeth and alveolar bones, along with restoration of periodontal tissue, and can produce bone cells and cement blast cells similar to osteoblasts [4,5]. Therefore, the efficacy of periodontitis treatment can be evaluated using the inflammatory state, proliferation, and osteogenic induction ability of HPDL cells.

The inflammasome is a vital signal mediated by the innate immune system, and is a group of multimeric cytoplasmic protein complexes consisting of associated speck-like protein containing a CARD (ASC) and caspase-1, and activation of caspase-1 by this com-plex causes the release of proinflammatory cytokines such as IL-1β and TNF-α [6]. The inflammasome, activated by metabolic impairment and infection, has also been suggested to be involved in periodontal disease pathogenesis [7]. It has been found that the mRNA expression of NLR family pyrin domain-containing 3 (NLRP3) and IL-1β is increased in patients with periodontitis [8]. cAMP-responsive element-binding protein (CREB) regulates cell proliferation, differentiation, and survival, along with inflammation in various cell types, and cAMP-dependent protein kinase A (PKA) has a variety of functions, including regulation of lipid metabolism in many kinds of cells [9,10]. Previous studies have shown that PKA regulates osteoblast-specific transcription factors such as Runt-related transcription factor 2 (RUNX2) and osterix. It has been reported that the cAMP/PKA/CREB axis can enhance bone formation in human mesenchymal stem cells [11,12]. However, the relationship and role of NLRP3 and cAMP/PKA/CREB axes in human periodontal ligament cells stimulated with PG-LPS have not been identified.

*Sargassum horneri* is a species of brown algae common along the coasts of Japan, China, and Korea. Growing to a length of approximately 7 m in sea areas with high water transparency and abundant nutrient salt [13], the seaweed has also recently settled off at the coast of Baja California in southern California and Mexico. Locally known as ‘*mojaban*’ in Korea, *S. horneri* has for centuries been used as an ingredient in traditional medicine to treat several diseases [14] and as source of food owing to its rich composition of amino acids, vitamins, and polysaccharides. It has been reported to have various physiological activities, such as anti-inflammatory, antiviral, antioxidant, and anti-cancer activities [15,16]. In addition, extracts of *S. horneri* have been reported to regulate immunomodulation and stress [17,18]. According to a recent study, it was found that *S. horneri* contains components such as (-)-loliolide, 3-hydroxy-5,6-epoxy-β-ionone (HEBI), and apo-9-fucoxanthinone, which are components of the norisoprenoid family and are known to have anti-inflammatory effects [14]. However, research on the bioactive components of *S. horneri* is still insufficient. Therefore, in this study, we evaluated the impact of compound epiloliolide isolated from *S. horneri* on the inhibition of inflammation and induction of osteoblast differentiation through cell proliferation, two critical therapeutic strategies for periodontitis, in human periodontal ligament cells stimulated with PG-LPS.

## 2. Results

### 2.1. Effects of Epiloliolide on Proliferation and Migration of Human Periodontal Ligament Cells

**Figure 1:**
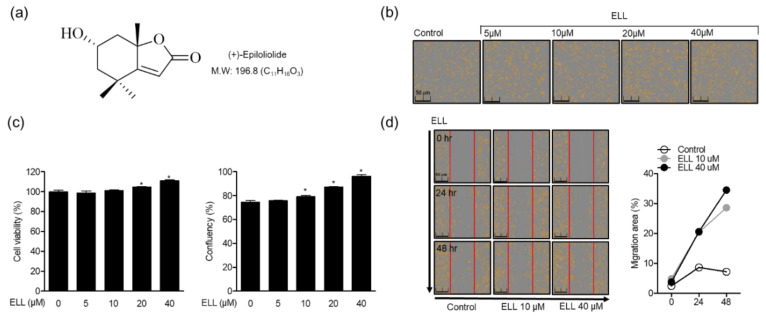
Effects of epiloliolide (ELL) on proliferation and migration of human periodontal ligament cells. (**a**) The chemical structure of ELL. (**b**) HPDL cells were treated with the indicated concentrations of epiloliolide for 24 h. (**c**) Then, cell viability was measured through MTT assay, and cell count was measured through Incucyte^®^ Live-Cell analysis systems. (**d**) The wound healing test was performed in HPDL cells with treatment of indicated concentrations of epiloliolide for 24 and 48 h, and the migration area (%) was quantified by Incucyte^®^ Live-Cell analysis systems. Data are presented as means ± SD of three independent experiments. * *p* < 0.05 was considered significant for differences between each group.

MTT and migration assays were performed to confirm the effect of epiloliolide (Figure 1a) on the toxicity and proliferation in human periodontal ligament (HPDL) cells. First, HPDL cells were treated with 5–40 μM of epiloliolide and cultured for 48 h. The results showed that epiloliolide induced cell proliferation in a concentration-dependent manner (Figure 1b). In addition, it was confirmed through MTT assay that there was no cell toxicity for 48 h after epiloliolide treatment of HPDL cells, and the confluence of cells was increased in a concentration-dependent manner (Figure 1c). Therefore, in this study, it was confirmed that the compound epiloliolide isolated from the *S. horneri* induces the proliferation of HPDL cells. As a result of performing a migration assay for 48 h to evaluate the mobility of the cells, epiloliolide increased cell migration in a time- and concentration-dependent manner (Figure 1d). These results suggest that epiloliolide affects not only the proliferation of HPDL cells but also the migration of cells.

### 2.2. Effect of Epiloliolide on Wound Healing and Osteoblast Differentiation in HPDL Cells Stimulated with PG-LPS

**Figure 2:**
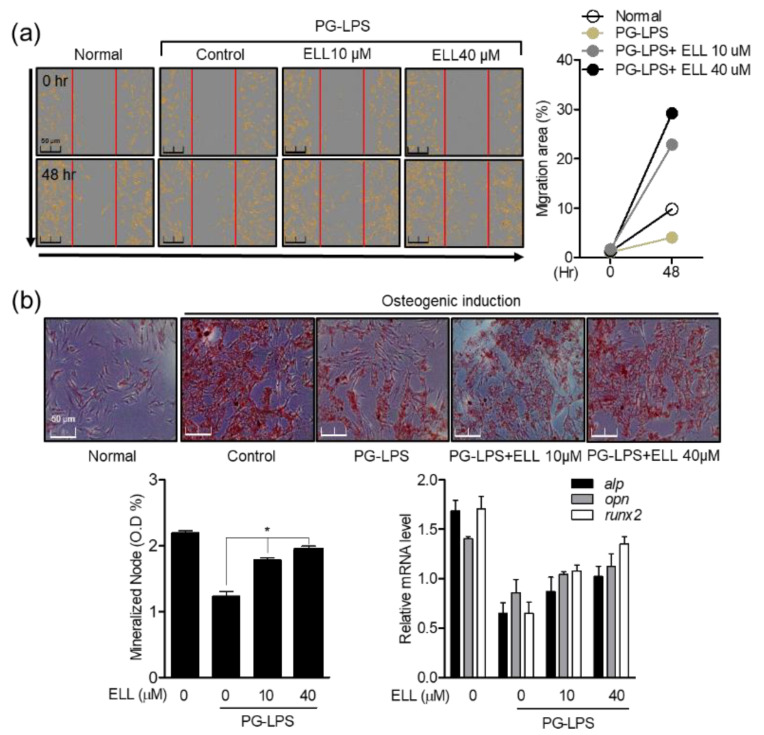
Effect of epiloliolide (ELL) on wound healing and osteoblast differentiation in HPDL cells stimulated with PG-LPS. (**a**) HPDL cells were pretreated with epiloliolide at the indicated concentration for 24 h and then treated with PG-LPS (1 μg/mL) for 48 h; then, cell migration area (%) was measured through Incucyte^®^ Live-Cell analysis systems. (**b**) The HPDL cells were pretreated with or without the indicated concentration of epiloliolide for 24 h, then incubated with PG-LPS for 14 days. The mineralization was measured by Alizarin Red S (ARS) staining. The level of osteogenic induction-specific genes *alp*, *opn*, and *runx2* was confirmed by real-time PCR. The results were normalized to *gapdh* expression. * *p* < 0.05 was considered significant for only PG-LPS treat group.

As described in Section 2.1, epiloliolide induced the proliferation and migration of HPDL cells without toxicity in a concentration-dependent manner. Therefore, the effect of epiloliolide on wound healing and osteoblast differentiation in HPDL cells stimulated with PG-LPS was evaluated. Epiloliolide also induced migration in HPDL cells stimulated with PG-LPS (Figure 2a), suggesting that epiloliolide may contribute to the restoration and protection of periodontal ligament cells, an important treatment strategy for periodontitis. Therefore, in this study, it was confirmed that epiloliolide induces not only the proliferation and migration of HPDL cells but also the differentiation of HPLD cells into osteoblasts. First, HPDL cells were cultured in osteogenic induction media for 14 days, and differentiation into osteoblasts was induced by treatment with PG-LPS alone or with epiloliolide. Alizarin Red S staining was performed to measure osteoblast differentiated mineralized nodes, and it was confirmed that the differentiation of osteoblasts inhibited by PG-LPS induced the differentiation of osteoblasts in the epiloliolide 10 μM treatment group. The effect was further enhanced in the 40 μM treatment group (Figure 2b). As a result, epiloliolide induced cell migration in HPDL cells stimulated with PG-LPS. This migration and proliferation inducing effect suggests that epiloliolide can play an essential role in alveolar bone loss, an important treatment strategy for periodontitis, even in the process of differentiation of HPDL cells into osteoblasts.

### 2.3. Epiloliolide Downregulates the Expression of NLRP3 Inflammasome and Pro-Inflammatory Mediators in HPDL Cells Stimulated with PG-LPS

**Figure 3:**
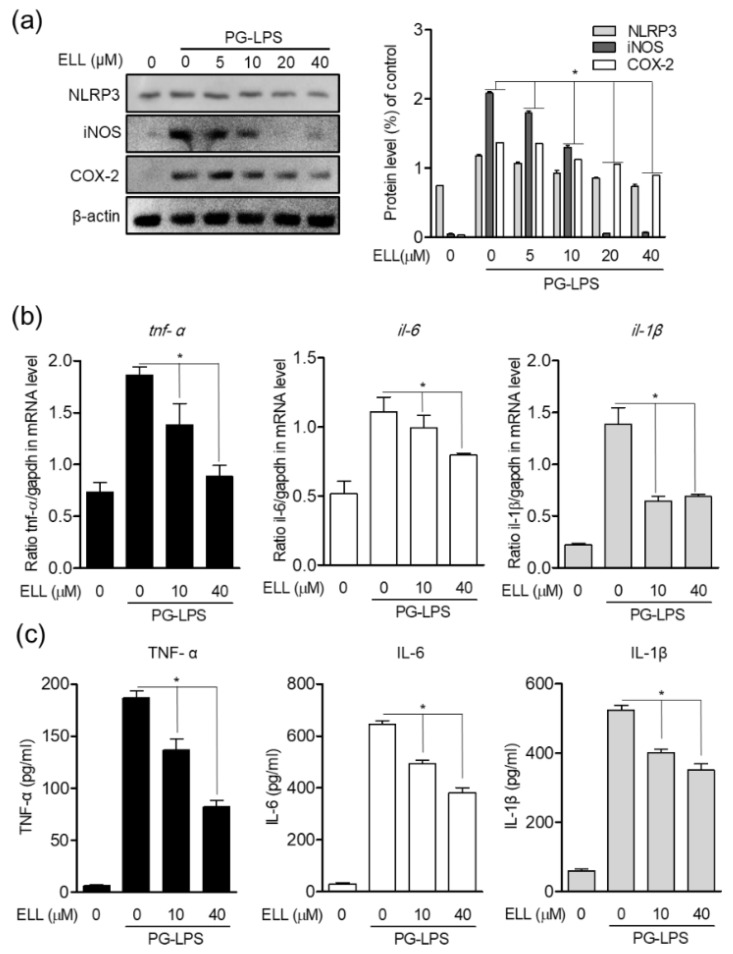
Epiloliolide (ELL) downregulates the expression of NLRP3 inflammasome and pro-inflammatory mediators in HPDL cells stimulated with PG-LPS. (**a**) Protein expression of NLRP3, iNOS, and COX-2 in HPDL cells pretreated with epiloliolide (0, 5, 10, 20, and 40 μM) for 24 h and then stimulated with PG-LPS (1 μg/mL) for 24 h was determined by Western blotting. The band shown intensities after normalization of the β-actin level. (**b**) The gene level of pro-inflammatory cytokines *il-6*, *tnf-α*, and *il-1β* were confirmed by real-time PCR. (**c**) The release of pro-inflammatory cytokines IL-6, TNF-α, and IL-1β were confirmed by ELISA assay. The results were normalized to *gapdh* expression. * *p* < 0.05 was considered significant for only PG-LPS treat group.

The NLRP3 inflammasome is activated by cell infection or stress and plays a vital role in the expression of proinflammatory mediators. Therefore, the effect of epiloliolide on the expression of proinflammatory mediators and cytokines according to the activity of the NLRP3 inflammasome in *Porphyromonas gingivalis* lipopolysaccharide (PG-LPS)-stimulated HPDL cells was evaluated. Epiloliolide increased the protein expression of NLRP3 inflammasome in HPDL cells stimulated with PG-LPS, and it was confirmed that the increased NLRP3 inflammasome was decreased in a concentration-dependent manner by treatment with epiloliolide. In addition, the activity of NLRP3 inflammasome by PG-LPS induced protein expression of proinflammatory mediators iNOS and COX-2 (Figure 3a), which inhibited the production of proinflammatory medium proteins and downregulated the gene levels of inflammatory cytokines such as *tnf-α*, *il-6*, and *il-1β* in a concentration dependent manner (Figure 3b). The release of these cytokines by PG-LPS was also inhibited (Figure 3c). These results suggest that epiloliolide can regulate the production of proinflammatory mediator proteins such as iNOS and COX-2 by inhibiting the activity of the NLRP3 inflammasome in PG-LPS-stimulated HPDL cells. In addition, it was confirmed that epiloliolide downregulated the gene expression of inflammatory cytokines, which play an important role in periodontitis.

### 2.4. Epiloliolide Upregulates Phosphorylation of the PKA/CREB Pathway

**Figure 4:**
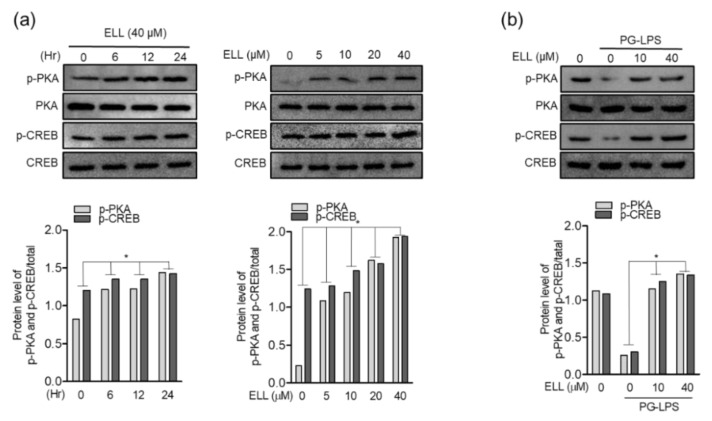
Epiloliolide (ELL) upregulates phosphorylation of the PKA/CREB pathway. (**a**) Western blot analysis of PKA (*p*-PKA)/CREB (*p*-CREB) expressions after treatment with indicated concentrations of epiloliolide (0, 5, 10, 20, and 40 μM) for 6, 12, or 24 h. (**b**) Protein expression of PKA (*p*-PKA) and CREB (*p*-CREB) in HPDL cells pretreated with epiloliolide (10 or 40 μM) for 24 h and then stimulated with PG-LPS (1 μg/mL) for 24 h was determined by Western blotting. The results were normalized to total protein expression. * *p* < 0.05 was considered significant for differences in control or only PG-LPS-treated groups.

Phosphorylation of the PKA/CREB protein is an important factor in regulating cell growth and proliferation. Therefore, it was confirmed through Western blot assay how phosphorylation of PKA/CREB protein is regulated in the HPDL cell proliferation effect of epiloliolide, as shown above. First, HPDL cells were treated with 40 μM of epiloliolide for 0, 6, 12, and 24 h to evaluate PKA/CREB protein phosphorylation. As a result, epiloliolide increased the phosphorylation of PKA/CREB protein in a time-dependent manner, and phosphorylation peaked 24 h after epiloliolide treatment. Next, the effect of concentration-dependent treatment with epiloliolide on the phosphorylation of PKA/CREB protein was evaluated. Phosphorylation of PKA/CREB protein was evaluated 24 h after treatment with 5, 10, 20, and 40 μM of epiloliolide, and phosphorylation was induced in a concentration-dependent manner (Figure 4a). In addition, it could be seen that epiloliolide again restores the PKA/CREB phosphorylation level inhibited by PG-LPS (Figure 4b). These results show that epiloliolide upregulated the phosphorylation of PKA/CREB protein in a time- and concentration-dependent manner in HPDL cells and restored the phosphorylation of PKA/CREB protein inhibited by PG-LPS, suggesting that the HPDL cell proliferation and migration effects of epiloliolide can be achieved through phosphorylation of PKA/CREB proteins.

### 2.5. Anti-Inflammatory Effect of Epiloliolide through the PKA/CREB Signaling Pathway

**Figure 5:**
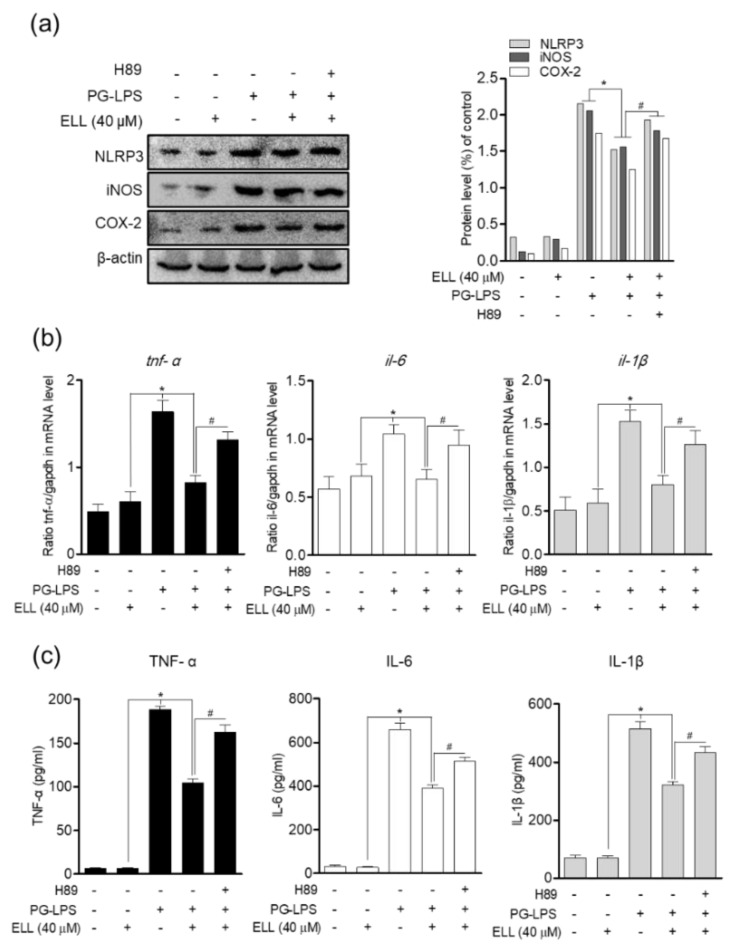
Anti-inflammatory effect of epiloliolide (ELL) through the PKA/CREB signaling pathway. (**a**) HPDL cells were treated with epiloliolide or PKA inhibitor H89 (20 μM) and then stimulated with PG-LPS for 24 h. The NLRP3, iNOS, and COX-2 protein expression in HPDL cells was analyzed by Western blot analysis, and the results were normalized to β-actin protein expression. (**b**) The gene level of proinflammatory cytokines *il-6*, *tnf-α*, and *il-1β* were confirmed by real-time PCR. (**c**) The release of proinflammatory cytokines IL-6, TNF-α, and IL-1β were confirmed by ELISA assay. The results were normalized to *gapdh* expression. * *p* < 0.05 was considered significant for only PG-LPS treat group. # *p* < 0.05 was considered significant for PG-LPS + ELL treat group.

Inhibition of the NLRP3 inflammasome activity has an important correlation with the migration and proliferation of cells by protecting cells by downregulating proinflammatory mediators. Therefore, in this study, the effect of treatment with H89, a PKA inhibitor, on the expression of NLRP3 inflammasome and proinflammatory mediators such as iNOS and COX-2 was evaluated. In HPDL cells, epiloliolide effectively downregulated the activity of the NLRP3 inflammasome activated by PG-LPS, as shown above, and thereby inhibited the expression of proinflammatory mediators iNOS and COX-2 proteins. However, as a result of inhibiting PKA signaling by treatment with the PKA inhibitor H89, it was confirmed that the protein expression of the proinflammatory mediator inhibited by epiloliolide was again upregulated (Figure 5a). In addition, treatment of H89 in HPDL cells stimulated with PG-LPS reversed the gene expression of the proinflammatory cytokines *tnf-α*, *il-6*, and *il-1β* inhibited by epiloliolide (Figure 5b). These results were similar for the release of inflammatory cytokines (Figure 5c). Therefore, it is proposed that the anti-inflammatory effect of epiloliolide in HPDL cells stimulated with PG-LPS, as described above, can be achieved through the regulation of PKA/CREB activity.

### 2.6. Epiloliolide Induces the Proliferation of HPDL Cells through the Regulation of Phosphorylation of the PKA/CREB Signaling Pathway

**Figure 6:**
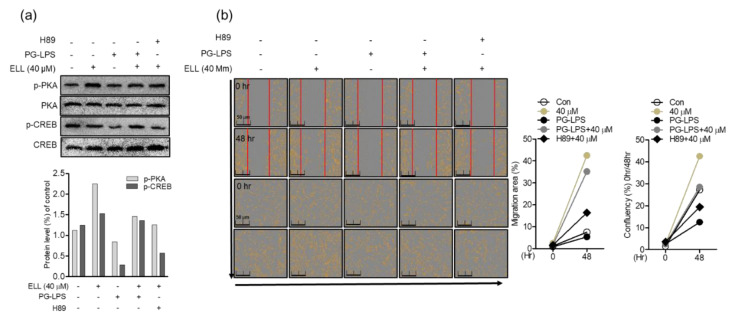
Epiloliolide (ELL) induces the proliferation of HPDL cells through the regulation of phosphorylation of the PKA/CREB signaling pathway. (**a**) HPDL cells were treated with epiloliolide or PKA inhibitor H89 (20 μM) and then stimulated with PG-LPS for 24 h. The PKA (*p*-PKA)/CREB (*p*-CREB) protein expression in HPDL cells was analyzed by Western blot analysis, and the results were normalized to β-actin protein expression. (**b**) HPDL cells were pretreated with epiloliolide or PKA inhibitor H89 (20 μM) at the indicated concentration for 24 h and then treated with PG-LPS (1 μg/mL) for 48 h, then the cell migration area and confluency (%) were measured through Incucyte^®^ Live-Cell analysis systems.

Induction of PKA/CREB activity by epiloliolide in HPDL cells stimulated with PG-LPS has been found to effectively inhibit the activity of NLRP3 inflammasome and proinflammatory mediators and cytokines. Therefore, in this study, the effect of epiloliolide induced PKA/CREB protein regulation on the proliferation of PG-LPS-stimulated HPDL cells was evaluated. As shown in the previous results, epiloliolide effectively recovered the phosphorylation of PKA/CREB lost to LPS, and this phosphorylation-inducing effect was reversed by treatment with the PKA inhibitor H89 (Figure 6a). Next, the effect of the induction of PKA/CREB by epiloliolide on the proliferation of HPLD cells was evaluated. Epiloliolide restored the proliferation of HPDL cells, whose proliferation was inhibited by PG-LPS. It was confirmed that this proliferative effect was inhibited by treatment with H89 (Figure 6b). These results suggest that in PG-LPS-stimulated HPDL cells, epiloliolide exhibits anti-inflammatory effects by modulating NLRP3 inflammasome activity and affects the proliferation of HPDL cells through regulation of PKA/CREB activity.

### 2.7. Epiloliolide Induces Osteoblast Differentiation of HPDL Cells through Regulation of the PKA/CREB Signaling Pathway

**Figure 7:**
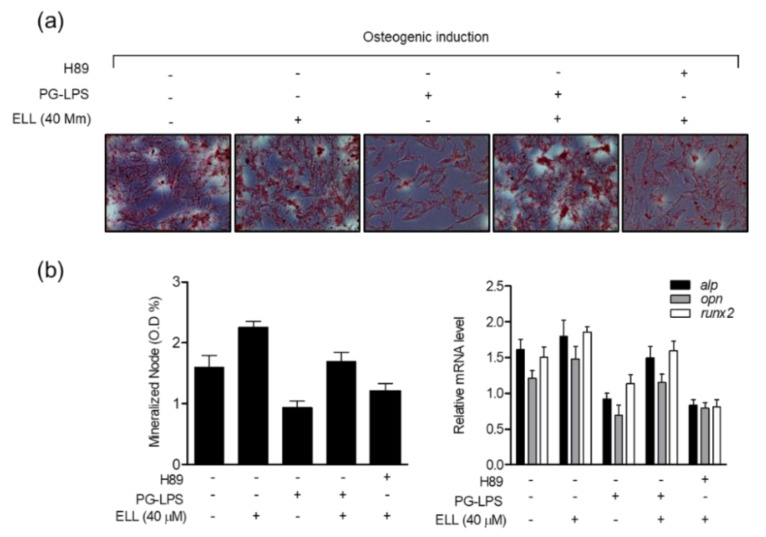
Epiloliolide (ELL) induces osteoblast differentiation of HPDL cells through regulation of the PKA/CREB signaling pathway. The HPDL cells were pretreated with or without the indicated concentration of epiloliolide or PKA inhibitor H89 (20 μM) for 24 h, then incubated with PG-LPS for 14 days. (**a**) The mineralization was measured by Alizarin Red S (ARS) staining. (**b**) The level of osteogenic induction-specific genes *alp*, *opn*, and *runx2* were confirmed by real-time PCR. The results were normalized to *gapdh* expression.

Previous studies have shown that epiloliolide regulates the anti-inflammatory activity and cell proliferation in PG-LPS-stimulated HPDL cells through PKA/CREB signaling. Therefore, to evaluate the effect of epiloliolide-induced PKA/CREB signaling on the differentiation process of HPDL cells into osteoblasts, the effect of the PKA inhibitor H89 treatment on osteoblast differentiation was evaluated. First, HPDL cells treated with or without H89 were cultured in an osteoblast differentiation induction medium and treated with or without epiloliolide and PG-LPS. As a result, epiloliolide differentiated HPDL cells into osteoblasts and restored the differentiation ability inhibited by PG-LPS. However, inhibition of the PKA/CREB signaling pathway by H89 treatment reversed the osteoblast inducing effect of epiloliolide-induced HPDL cells (Figure 7a). In addition, epiloliolide restored the levels of osteoblast-specific genes such as *alp*, *opn*, and *runx2* lost by PG-LPS during the osteoblast differentiation process of HPDL cells, and this effect was reversed by H89 treatment (Figure 7b). These results suggest that epiloliolide not only regulates inflammation and cell proliferation through the PKA/CREB signaling pathway but also participates in the differentiation of HPDL cells into osteoblasts.

## 3. Discussion

Interest and research in marine natural products continues to this day, and the development of healthy functional foods and pharmacological studies have been conducted [19]. Various pharmacological effects of marine-derived natural products, such as anti-inflammatory, anti-cancer, wound healing, and antioxidant activities, have been proven [20,21,22]. However, further research needs to be conducted to clarify the components, physiology, and pharmacological properties of natural products derived from the ocean that are still unknown. Epiloliolide ((6S, 7aS)-6-hydroxy-4,4,7a-trimethyl-5,6,7,7a-tetrahydrobenzofuran-2(4H)-one) is a monoterpene lactone derived from the degradation of carotenoids. It occurs as the stereoisomer of the more abundant lactone loliolide [23,24]. According to Isoe et al. [25], loliolide and epiloliolide are photo-oxidation products of algal carotenoids such as fucoxanthin and zeaxanthin. The occurrence of loliolide in a wide range of organisms, including plants, insects, and marine algae, has been documented [26]. Its stereoisomer epiloliolide has also been isolated from various brown algae and Sargassum species [27,28]. The presence of loliolide in *S. horneri* was first reported by Kim et al. [14]. However, the occurrence of epiloliolide in *S. horneri* has not been previously reported. Studies have shown the inhibition of germination [29], anti-hepatocellular carcinoma [30], and anti-melanogenesis [31] activities of epiloliolide. Despite the confirmed presence of epiloliolide in several species, few studies have investigated the potential bioactive properties of this norisoprenoid. Therefore, this study demonstrated that the compound epiloliolide isolated from *S. horneri* induces cell proliferation and osteoblast differentiation through anti-inflammatory effects in human periodontal ligament (HPDL) cells.

The activity of NLRP3 inflammasome by PG-LPS destroys the periodontal ligament by inducing a periodontal inflammatory reaction and inhibiting proinflammatory cyto-kines such as IL-1β, IL-6, and TNF-α against these pathogen infections, and mediators have been important in the treatment of periodontitis [32,33]. In addition, according to a recent study, it was found that the expression of IL-1β and NLRP3 inflammasome was higher in gingival tissue of periodontitis than in normal tissue [34]. In this study, epiloliolide effectively downregulated activated NLRP3 in HPDL cells stimulated with PG-LPS, thereby inhibiting protein expression of proinflammatory mediators such as iNOS and COX-2. At the same time, the levels of inflammatory cytokines were also downregulated. These results suggest that the previously reported periodontal ligament fibroblasts and periodontal ligament cells can regulate the inflammatory response by inhibiting NLRP3 inflammasome activity [35]. The PKA/CREB signaling pathway is involved in the growth and proliferation of various cells, and phosphorylation of PKA/CREB proteins has been reported to promote the differentiation ability of osteoblasts and the expression of osteoblast-specific genes, such as bone morphogenetic protein 2 (BMP2), RUNX2, OCN, and ALP, through the phosphorylation of PKA/CREB protein [36,37]. In the present study, the phosphorylation of PKA/CREB protein increased with time and concentration by treatment with epiloliolide, and epiloliolide recovered the phosphorylation of suppressed PKA/CREB protein in HPDL cells stimulated with PG-LPS and promoted cell proliferation. In addition, epiloliolide restored the osteoblast differentiation ability lost by PG-LPS during the osteoblast differentiation process of HPDL cells and upregulated the levels of osteoblast differentiation, inducing specific genes such as *alp*, *opn*, and *runx2*. According to previous research reports, apoptosis and cytoprotective effects through inhibition of NLRP3 activity in human osteoblasts such as MG63 are already known [38,39]. However, the role of NLRP3 in osteoblast differentiation of HPDL cells has not been identified.

In this study, the NLRP3 inflammasome and proinflammatory mediators and cytokines were inhibited through the induction and regulation of phosphorylation of PKA/CREB protein by epiloliolide. In addition, epiloliolide promoted the differentiation of HPDL cells into osteoblasts through the cell proliferation effect, and it was confirmed that the PKA/CREB signal regulation effect of epiloliolide is involved in osteoblast differentiation. These effects suggest the potential of epiloliolide as a therapeutic agent for periodontitis for the restoration of lost periodontal ligaments and osteoblasts, along with inhibition of inflammation, two important therapeutic strategies for periodontitis.

## 4. Materials and Methods

### 4.1. Chemicals and Reagents

Dulbecco’s modified Eagle’s medium (DMEM), minimum essential medium alpha (α-MEM), and fetal bovine serum (FBS) were purchased from Welgene Bioscience (Daegu, Korea). Trypsin-ethylene diamine tetra acetic acid (EDTA) culture reagents and penicillin were obtained from Gibco (Grand Island, NY, USA). 3-(4,5-Dimethylthiazol-2-yl)-2,5-diphenyltetrazoliumbromide (MTT), Alizarin Red S, and Dimethyl Sulfoxide-Dimethyl Sulfoxide (DMSO) were purchased from Sigma-Aldrich (Saint Louis, MO, USA). Lipopolysaccharide isolated from *P. gingivalis* (PG-LPS) was purchased from Invivo Gen (San Diego, CA, USA). iNOS, COX-2, and β-actin primary antibodies used for Western blot analysis were purchased from Santa Cruz Biotechnology Inc. (Dallas, TX, USA). Phosphorylated CREB, CREB, and NLRP3 were obtained from Cell Signaling Technology (Danvers, MA, USA), and phosphorylated PKA and PKA were purchased from Abcam (Cambridge, UK). In addition, secondary mouse and rabbit monoclonal antibodies were purchased from Santa Cruz Biotechnology Inc. Analytical grade solvents used for extraction and fractionation were supplied by Duksan pure chemicals Co. (Ansan, Korea). HPLC grade acetonitrile (ACN) and water were purchased from J.T. Baker (Philipsburg, NJ, USA). Triflouroacetic acid (TFA) was purchased from Sigma-Aldrich (St. Louis, MO, USA). Solvents used for NMR were purchased from Duchefa Biochemie (Haarlem, The Netherlands).

### 4.2. Plant Materials

*S. horneri* samples were collected from the north-western shores of Jeju Island, Korea, in May 2020. The algal specimen was kept at −20 °C until it was investigated.

### 4.3. Extraction and Isolation

The frozen specimen of *S. horneri* (3.0 kg) was lyophilized, and the dried algal sample (344 g) was extracted with 7 L of a mixture of dichloromethane and methanol (DCM:MeOH, 1:1) 7 times successively, and the filtrate was concentrated in a vacuum to yield 20.63 g of crude extract that was suspended in MeOH and extracted with n-hexane. The methanol portion was separated and concentrated, then suspended in water followed by sequential partitioning between EtOAc and butanol to obtain 4.03, 8.25, and 2.8 g, respectively. The EtOAc fraction was subjected to size exclusion chromatography on a Sephadex LH-20 column and eluted with DCM:MeOH (1:1), yielding 5 fractions as follows: 1 (2193.8 mg), 2 (1973.8 mg), 3 (648.7 mg), 4 (266.6 mg), and 5 (327.8 mg). EtOAc fraction number 4 was further fractionated by reverse phase chromatography using a C18 preparative column (Hector C18-M510, 16H1001, 250 × 21.2 mm), using a gradient elution system of 10% ACN for 5 min, and 10–100% ACN (5–60 min) with acetonitrile and 0.1% TFA in HPLC grade water as the mobile phase at a flow rate of 10 mL/min. The ultraviolent absorbance was observed at 220 and 250 nm by a Waters 2489 UV/Visible detector (Milford, MA, USA) coupled with a binary HPLC pump (Waters 1525, Milford, MA, USA). The isolated compound (2.7 mg) was eluted at 25.55 min with 95% purity.

### 4.4. Structural Identification of the Compound

The molecular weight of the compound was analyzed by an electrospray ionization (ESI)-triple quadrupole mass spectrometer API2000 (AB Sciex, Foster City, CA, USA). The compound was dissolved in deuterated chloroform (CDCl_3_) and its ^1^H and ^13^C spectra data were recorded on an Ascend^TM^ 500 spectrometer (Bruker, Billerica, MA, USA). With consistent similarity of the spectra data to that reported by references [27,40], the compound was confirmed as (+)-epiloliolide (ELL). The identification of structure using NMR is as follows:

(6S, 7aS)-6-hydroxy-4,4,7a-trimethyl-5,6,7,7a-tetrahydrobenzofuran-2(4H)-one, (+)-epiloliolide: a colorless gum; ESI-MS *m*/*z* 196.8 [M + H] +, C_11_H_16_O_3_ ^1^H-NMR (500 MHz, CDCl_3_) δ: 1.25 (3H, s, H-10), 1.29 (3H, s, H-9), 1.31 (1H, dd, *J* = 12.1 Hz, H-5), 1.49 (2H, t, *J* = 11.9, 11.7 Hz, H-7), 1.56 (3H, s, H-8), 1.56 (1H, s, OH), 2.01 (1H, ddd, *J* = 2.0, 6.6 and 12.8 Hz, H-5), 2.53 (1H, ddd, *J* = 1.8, 6.3 and 11.8 Hz, H-7), 4.11 (1H, tt, *J* = 4.2 and 11.7 Hz, H-6) and 5.69 (1H, s, H-3). ^13^C- NMR (500 MHz, CDCl_3_) δ: 25.31 (C-10), 25.82 (C-9), 30.15 (C-8), 35.27 (C-4), 48.14 (C-7), 50.06 (C-5), 65.31 (C-6), 86.61 (C-7a), 113.53 (C-3), 171.81 (C-2), 180.86 (C-3a).

### 4.5. Preparation and Culture of Human Periodontal Ligament Cells

HPDL cells were obtained from the third molars of each donor as previously described [41], and the protocol for the isolation and cultivation of cells was reviewed and approved by the Institutional Review Board (KNU 2017–78) of Kyungpook National University (Daegu, Korea). The HPDL cells were cultured in α-MEM supplemented with 10% (*v*/*v*) fetal bovine serum (FBS) and 1% penicillin/streptomycin, then cultured at 37 °C in a humidified atmosphere with 5% CO_2_.

### 4.6. MTT and Coefficient Assays

In order to evaluate the cytotoxicity of epiloliolide in HPDL cells, after culturing HPDL (5 × 10^3^ cells/mL) in a 96-well plate for 24 h, then after, 5, 10, 20, and 40 μM of epiloliolide were treated for 24 h. Thereafter, cell counts were measured using the Incucyte^®^ Live-Cell assay system (Göttingen, Germany), and cytotoxicity was evaluated by the MTT (3-[4,5-dimethylthiazol-2-yl]-2,5-diphenyl tetrazolium bromide) assay on HPDL cells. For MTT analysis, 100 µL of 5 mg/mL MTT solution was treated for 4 h, the medium was removed, and 200 µL of dimethyl sulfoxide (DMSO) was added. Then, the absorbance was measured at a 540 nm wavelength using a microplate reader (Tecan Trading AG) (Männedorf, Switzerland).

### 4.7. Wound Healing Assays

The mobility of HPDL cells by epiloliolide was evaluated through the scratch test. The HPDL cells were seeded into a 12-well plate (5 × 10^5^ cells/well), and cultured for 24 h. Then, the cells were scraped vertically with a 200 μL pipette tip, and the cells were washed three times with PBS and then treated with epiloliolide (10 and 40 μM), and incubated for 48 h. Images were taken at 24 and 48 h respectively, through Incucyte^®^ Live-Cell analysis systems, and the area of cells moved from the baseline of the wound was measured and evaluated.

### 4.8. Mineralization Assay

HPDL cells were seeded at 1 × 10^4^ cells/well in a 6-well plate and then prepared for osteogenic induction. Next, cells were cultured in osteo induction medium (OIM) containing 50 μg/mL of ascorbic acid, 0.1 μM of dexamethasone, and 10 mM of β-glycerophosphate for 14 days. During the osteogenic induction period, the indicated concentrations of PG-LPS or epiloliolide were treated and cultured. The mineralized nodules were formed, and the mineralized cells were fixed with 4% polyformaldehyde for 30 min, stained with 0.1% Alizarin Red S, and kept at room temperature at pH 4.3 for 30 min. To measure the content of calcium precipitate, the cetyl pyridine chloride (CPC) method was applied, and the absorbance at 560 nm was measured using a multifunctional microplate reader (M1000 Pro, TECAN, Männedorf, Switzerland).

### 4.9. Western Blot Analysis

For Western blot analysis, RIPA lysis buffer (Sigma-Aldrich, USA) containing 50 mM Tris pH 8.0, 150 nM NaCl, 0.02% sodium azide, 0.2% SDS, 1 M MPMFS, 10 μL/mL aprotinin, 1% igapel 630, 10 nM NaF, and 0.5 nM EDTA was used to harvest proteins from HPDL cells. Quantification of protein concentration for each sample was determined using a BCA protein assay kit (Thermo Fisher Scientific, MA, USA), and the quantified sample was separated by 10–15% SDS-PAGE gel electrophoresis at 20 or 30 μg and electrolyzed on ice to obtain polyvinylidene fluoride (PVDF) transferred to membranes. Then, for blocking the PVDF membrane, it was shaken for 45 min with Tris-buffered saline with 0.1% Tween^®^ 20 detergent (TBST), supplemented with 5% BSA, and then washed 3 times for 10 min each. Next, the primary antibody against the target protein was reacted at 4 degrees for 24 h, washed 3 times with TBST for 10 min, and then the secondary antibody was reacted for 2 h. For detection of Western blots, proteins were activated using an enhanced chemiluminescence (ECL) reagent (Thermo Fisher Scientific, MA, USA) and analyzed using an Image Quant LAS 4000 bio-molecular imager (GE Healthcare Life Sciences, Marlborough, MA, USA).

### 4.10. Quantitative Real-Time PCR Analysis

qRT-PCR was used to analyze the mRNA levels of *il-1β*, *il-6*, *tnf-α*, *alp*, *opn*, *runx2,* and *gapdh* genes. Total RNA was extracted from HPDL cells using TRIzol/chloroform reagent (Bioneer, Daejeon, Korea) according to the manufacturer’s instructions, and reverse transcribed into cDNA using the PrimeScript-RT reagent kit. Thereafter, cDNA was amplified by SYBR Premix Ex Taq (Takara, Japan), including each gene-specific primer. Then, *gapdh* was used as the housekeeping gene. The cycle threshold (Ct) values of the target genes were normalized to *gapdh*. The primers for each gene used in this study were supplied by Biomedic Co. (Bucheon, Korea) and the sequences of primers are shown in Table 1. The qRT-PCR analysis result was calculated using the following equation: 2 − ΔΔCT, where ΔΔCT = (CT*target* − CT*gapdh*) at time x − (CT*target* − CT*gapdh*) at time 0, where time x represents any time points, and time 0 represents the 1× expression of the gene in each group normalized to *gapdh*.

### 4.11. Statistical Analysis

Independent sample statistical analysis was performed between analyses with respect to the mean ± standard deviation (SD) for the triplicated experiments using SPSS statistics 19.0 software (Armonk, NY, USA). Differences between groups were analyzed by one-way analysis of variance (ANOVA) followed by Tukey’s test or Student’s *t*-test. *p* < 0.05 was considered statistically significant.

## 5. Conclusions

This study investigated the anti-inflammatory and proliferation-inducing effects of epiloliolide in HPDL cells stimulated with PG-LPS by controlling the NLRP3 inflammasome through PKA/CREB, and evaluated its effect on osteoblast differentiation of HPDL cells. The correlation between NLRP3 inflammasome and PKA/CREB in osteoblasts such as MG63 has been reported, but this is the first study result reported in HPDL cells. This study proposes the possibility of using epiloliolide as a fundamental treatment for periodontitis.

## Figures and Tables

**Table 1:** Primer sequences.

Target Gene	Sequence (5′→3′)	
*il-6*	Forward	AGTGAGGAACAAGCCAGAGC
	Reverse	GTCAGGGGTGGTTATTGCAT
*il-1β*	Forward	AACCTCTTCGAGGCACAAGG
	Reverse	GTCCTGGAAGGAGCACTTCAT
*tnf-α*	Forward	GCCTCTTCTCCTTCCTGATCGT
	Reverse	TGAGGGTTTGCTACAACATGGG
*alp*	Forward	TGCAGTACGAGCTGAACAGG
	Reverse	GTCAATTCTGCCTCCTTCCA
*opn*	Forward	TCAGCTGGATGACCAGAGTG
	Reverse	TTGGGGTCTACAACCAGCAT
*runx2*	Forward	TCTTAGAACAAATTCTGCCCTTT
	Reverse	TGCTTTGGTCTTGAAATCACA
*gapdh*	Forward	TGTTCGTCATGGGTGTGAAC
	Reverse	GTCTTCTGGGTGGCAGTGAT

## Data Availability

Data sharing is not applicable to this article.

## References

[1] Graves D.T., Oskoui M., Volejnikova S., Naguib G., Cai S., Desta T., Kakouras A., Jiang Y. (2001). Tumor necrosis factor modulates fibroblast apoptosis, PMN recruitment, and osteoclast formation in response to P. gingivalis infection. J. Dent. Res..

[2] Graves D., Cochran D. (2003). The Contribution of Interleukin-1 and Tumor Necrosis Factor to Periodontal Tissue Destruction. J. Periodontol..

[3] Kuboniwa M., Lamont R.J. (2009). Subgingival biofilm formation. Periodontol. 2000.

[4] Uchiyama M., Nakamichi Y., Nakamura M., Kinugawa S., Yamada H., Udagawa N., Miyazawa H. (2009). Dental Pulp and Periodontal Ligament Cells Support Osteoclastic Differentiation. J. Dent. Res..

[5] Cho M.-I., Garant P.R. (2000). Development and general structure of the periodontium. Periodontol. 2000.

[6] Van Opdenbosch N., Gurung P., Walle L.V., Fossoul A., Kanneganti T.-D., Lamkanfi M. (2014). Activation of the NLRP1b inflammasome independently of ASC-mediated caspase-1 autoproteolysis and speck formation. Nat. Commun..

[7] Huang X., Yang X., Ni J., Xie B., Liu Y., Xuan D., Zhang J. (2015). Hyperglucose Contributes to Periodontitis: Involvement of the NLRP3 Pathway by Engaging the Innate Immunity of Oral Gingival Epithelium. J. Periodontol..

[8] Bostanci N., Emingil G., Saygan B., Turkoglu O., Atilla G., Curtis M.A., Belibasakis G.N. (2009). Expression and regulation of the NALP3 inflammasome complex in periodontal diseases. Clin. Exp. Immunol..

[9] Pasqualucci L., Kitaura Y., Gu H., Dalla-Favera R. (2006). PKA-mediated phosphorylation regulates the function of activation-induced deaminase (AID) in B cells. Proc. Natl. Acad. Sci. USA.

[10] Skalhegg B.S., Tasken K. (2000). Specificity in the cAMP/PKA signaling pathway. Differential expression, regulation, and subcellular localization of subunits of PKA. Front. Biosci..

[11] Lo K.W.-H., Kan H.M., Ashe K.M., Laurencin C.T. (2011). The small molecule PKA-specific cyclic AMP analogue as an inducer of osteoblast-like cells differentiation and mineralization. J. Tissue Eng. Regen. Med..

[12] Siddappa R., Martens A., Doorn J., Leusink A., Olivo C., Licht R., van Rijn L., Gaspar C., Fodde R., Janssen F. (2008). cAMP/PKA pathway activation in human mesenchymal stem cells in vitro results in robust bone formation in vivo. Proc. Natl. Acad. Sci. USA.

[13] Umezaki I. (1984). Ecological studies of *Sargassum horneri* (TURNER) C. AGARDH in Obama Bay, Japan Sea. Nippon. Suisan Gakkaishi.

[14] Kim H.S., Priyan I., Fernando S., Lee S.H., Ko S.C., Kang M.C., Ahn G., Je J.G., Shin H.J., Lee W.W. (2021). Isolation and characterization of anti-inflammatory compounds from Sargassum horneri via high-performance centrifugal partition chromatography and high-performance liquid chromatograph. Algal. Res..

[15] Shao P., Chen X., Sun P. (2014). Chemical characterization, antioxidant and antitumor activity of sulfated polysaccharide from Sargassum horneri. Carbohydr. Polym..

[16] Sanjeewa K.K., Fernando I.P., Kim E.A., Ahn G., Jee Y., Jeon Y.J. (2017). Anti-inflammatory activity of a sulfated polysaccharide isolated from an enzymatic digest of brown seaweed Sargassum horneri in RAW 264.7 cells. Nutr. Res. Pract..

[17] Kim D.-S., Sung N.-Y., Park S.-Y., Kim G., Eom J., Yoo J.-G., Seo I.-R., Han I.-J., Cho Y.-B., Kim K.-A. (2018). Immunomodulating activity of Sargassum horneri extracts in RAW264.7 macrophages. J. Nutr. Health.

[18] Park S., Thomas S.S., Cha Y.S., Kim K.A. (2020). Inhibitory effects of *Sargassum horneri* extract against endoplasmic reticulum stress in HepG2 cells. J. Nutr. Health.

[19] Lee B.J. (2013). Development of Functional Food Using Fermented Marine Organism. Food Sci. Nutr..

[20] Yu T.J., Cheng Y.B., Lin L.C., Tsai Y.H., Yao B.Y., Tang J.Y., Chang F.R., Yen C.H., Ou-Yang F., Chang H.W. (2021). Physalis peruviana-Derived Physapruin A (PHA) Inhibits Breast Cancer Cell Proliferation and Induces Oxidative-Stress-Mediated Apoptosis and DNA Damage. Antioxidants.

[21] Lin H., Zheng Z., Yuan J., Zhang C., Cao W., Qin X. (2021). Collagen Peptides Derived from *Sipunculus nudus* Accelerate Wound Healing. Molecules.

[22] Pradhan B., Patra S., Behera C., Nayak R., Jit B., Ragusa A., Jena M. (2021). Preliminary Investigation of the Antioxidant, Anti-Diabetic, and Anti-Inflammatory Activity of *Enteromorpha intestinalis* Extracts. Molecules.

[23] Percot A., Yalcin A., Aysel V., Erdugan H., Dural B., Guven K.C., Yalçın A., Güven K.C. (2009). Loliolide in marine algae. Nat. Prod. Res..

[24] Mori K., Khlebnikov V. (1993). Carotenoids and Degraded Carotenoids, VIII–Synthesis of (+)-Dihydroactinidiolide,(+)-and (−)-Actinidiolide,(+)-and (−)-Loliolide as well as (+)-and (−)-Epiloliolide. Liebigs Annalen Chemie.

[25] Isoe S., Hyeon S.B., Katsumura S., Sakan T. (1972). Photo-oxygenation of carotenoids. II. The absolute configuration of loliolide and dihydroactinidiolide. Tetrahedron Lett..

[26] Grabarczyk M., Wińska K., Mączka W., Potaniec B., Anioł M. (2015). Loliolide—The most ubiquitous lactone. Folia Biol. Oecologica.

[27] Park K.-E., Kim Y.A., Jung H.A., Lee H.-J., Ahn J.-W., Lee B., Seo Y. (2004). Three Norisoprenoids compounds isolated from brown algae insects. J. Korean Chem. Soc..

[28] Peng Y., Huang R.-M., Lin X.-P., Liu Y.-H. (2018). Norisoprenoids from the Brown Alga *Sargassum naozhouense* Tseng et Lu. Molecules.

[29] Kuniyoshi M. (1985). Germination Inhibitors from the Brown Alga *Sargassum crassifolium* (Phaeophyta, Sargassaceae). Bot. Mar..

[30] Gangadhar K.N., Rodrigues M.J., Pereira H., Gaspar H., Malcata F.X., Barreira L., Varela J. (2020). Anti-Hepatocellular Carci-noma (HepG2) Activities of Monoterpene Hydroxy Lactones Isolated from the Marine Microalga Tisochrysis Lutea. Mar. Drugs.

[31] Ko R.K., Kang M.-C., Kim S.S., Oh T.H., Kim G.-O., Hyun C.-G., Hyun J.W., Lee N.H. (2013). Anti-melanogenesis Constituents from the Seaweed Dictyota Coriacea. Nat. Prod. Commun..

[32] Schenkein H.A. (2006). Host responses in maintaining periodontal health and determining periodontal disease. Periodontol. 2000.

[33] Abais J.M., Xia M., Zhang Y., Boini K.M., Li P.-L. (2015). Redox Regulation of NLRP3 Inflammasomes: ROS as Trigger or Effector?. Antioxid. Redox Signal..

[34] Bostanci N., Belibasakis G.N. (2012). *Porphyromonas gingivalis*: An invasive and evasive opportunistic oral pathogen. FEMS Microbiol. Lett..

[35] Lian D., Daia L., Xie Z., Zhou X., Liu X., Zhang Y., Huang Y., Chen Y. (2018). Periodontal ligament fibroblasts migration injury via ROS/TXNIP/Nlrp3 inflammasome pathway with *Porphyromonas gingivalis* lipopolysaccharide. Mol. Immunol..

[36] Li J., Hao L., Wu J., Zhang J., Su J. (2016). Linarin promotes osteogenic differentiation by activating the BMP-2/RUNX2 pathway via protein kinase A signaling. Int. J. Mol. Med..

[37] Chen M., Cui Y., Li H., Luan J., Zhou X., Han J. (2019). Icariin Promotes the Osteogenic Action of BMP2 by Activating the cAMP Signaling Pathway. Molecules.

[38] Xu L., Zhang L., Wang Z., Li C., Li S., Li L., Fan Q., Zheng L. (2018). Melatonin Suppresses Estrogen Deficiency-Induced Os-teoporosis and Promotes Osteoblastogenesis by Inactivating the NLRP3 Inflammasome. Calcif. Tissue Int..

[39] Ran S., Chu M., Gu S., Wang J., Liang J. (2019). Enterococcus faecalis induces apoptosis and pyroptosis of human osteoblastic MG63 cells via the NLRP3 inflammasome. Int. Endod. J..

[40] Kimura J., Maki N. (2002). New Loliolide Derivatives from the Brown Alga Undaria pinnatifida. J. Nat. Prod..

[41] Seo B.M., Miura M., Gronthos S., Bartold P.M., Batouli S., Brahim J., Young M., Robey P.G., Wang C.Y., Shi S. (2004). Inves-tigation of multipotent postnatal stem cells from human periodontal ligament. Lancet.

